# Clinical Validity of Wrist‐ and Trunk‐Worn Sensor‐Derived Data Models in Parkinson’s Disease

**DOI:** 10.1155/padi/8598501

**Published:** 2026-08-07

**Authors:** Yanke Sun, Sally Day, Kate Walters, Anette Schrag

**Affiliations:** ^1^ Department of Clinical Neurosciences, UCL Queen Square Institute of Neurology, University College London, London, UK, ucl.ac.uk; ^2^ Department of Electronic and Electrical Engineering, University College London, London, UK, ucl.ac.uk; ^3^ Department of Primary Care and Population Health, University College London, London, UK, ucl.ac.uk

**Keywords:** digital tools, health monitoring, machine learning, Parkinson’s disease, validity, wearables

## Abstract

**Background:**

Clinical rating scales for Parkinson’s disease (PD) have limitations in the accurate assessment of disease severity, which may obscure treatment effects in clinical management and trials. Body‐worn sensors can provide data for continuous and more precise monitoring of motor features of PD in patients’ daily lives. However, little information exists on the clinical validity of sensor‐derived data.

**Objectives:**

We assessed the clinical validity of outputs from three different machine learning models using trunk‐ or wrist‐worn sensors in patients with PD, assessing their correlations with scores on clinical scales assessing motor severity and impact on function.

**Methods:**

Wrist‐ and/or trunk‐worn sensors were worn by patients with PD, who had been assessed using the MDS‐UPDRS and the EQ‐5D‐5L, for up to one week. Output data were analyzed using three different algorithms: One trained on a publicly available dataset using trunk sensor data and two previously derived from wrist sensor data. Clinical validity was examined by examining correlations of sensor‐derived outputs with individual items of the MDS‐UPDRS and EQ‐5D‐5L.

**Results:**

For the trunk‐worn sensor‐derived outputs, the strongest positive correlations were found between output data and axial features such as arising from a chair, posture, and body bradykinesia and aspects of daily functioning on the MDS‐UPDRS Part II and health‐related quality of life (EQ‐5D‐5L domain) scores. For the wrist‐worn sensor‐derived outputs, the strongest positive correlations were seen between output data and postural tremor, rest tremor amplitude, and ability to undertake hobbies. Outputs from both body locations were correlated with MDS‐UPDRS II and EQ‐5D‐5L scores (*r* > 0.7). In participants who wore both trunk and wrist sensors, percentage of time spent in different activities was similar between trunk‐ and wrist‐worn devices, except for time spent “Lying down” derived from the trunk‐worn sensor compared to time spent “Sleeping” derived from the wrist‐worn sensor algorithm.

**Conclusion:**

These data provide preliminary evidence for the clinical validity of single sensor assessments as measures of severity of motor features and motor functioning in patients with PD for use in clinical trials and practice. The results should be confirmed in large and more diverse populations and expanded to include other assessment methods such as laboratory‐based motor measurements.

## 1. Introduction

The assessment of the severity of Parkinson’s disease (PD) and its individual features is typically undertaken using clinical rating scales, such as the Movement Disorder Society–Unified Parkinson’s Disease Rating Scale (MDS‐UPDRS) motor part, at specific time points [1]. This has allowed for standardized assessments in clinical trials and comparison of results between studies in PD across populations, settings, modalities, and methodologies, can be applied without additional equipment, and is widely accepted as the gold standard of assessment. There are established training programs for raters that provide good interrater reliability. However, clinical ratings remain subject to some rater subjectivity, selection of features, and are limited by grouping into ordinal data and, most importantly, the necessity to evaluate clinical findings at a single point in time. In a disorder in which clinical features can vary considerably, particularly with advancing disease and in those with fluctuations in response to medication, or simply due to fatigue or other confounders, such assessments may not capture the full picture of disease severity, even in optimally controlled conditions. Body‐worn sensors can provide data for continuous monitoring of motor features of PD and assessment of function in patients’ daily lives [2–5]. They may provide more objective information on aspects of disease severity and capture these over prolonged periods of time during patients’ day‐to‐day lives. Several studies, including clinical trials, have utilized this potential and reported correlations with motor features [6, 7] and greater sensitivity to change than clinical outcomes [8, 9]. However, significant challenges remain in this field due to variations in devices, sensor placement, analytic algorithms (often not publicly available), and output variables, with limited clinical validation data available [10, 11]. In addition, current digital outcomes have predominantly been developed to capture isolated constructs of interest (e. g., tremor, bradykinesia or dyskinesia), failing to provide a comprehensive clinical picture or focus on patient‐centered outcomes [7, 12, 13]. Additionally, most studies evaluating wearable devices are conducted in controlled laboratory environments using specific tasks, and only a few high‐quality studies are performed in real‐world settings [5, 14], largely due to the challenges associated with data collection, technical validation, and the establishment of clinical gold standards for comparison [15]. Furthermore, available studies used different methodologies and populations, making comparisons difficult. In recent years, the public availability of algorithms for analysis of body‐worn sensor data has made it possible to undertake more standardized assessments and comparisons [16, 17]. However, to date, very few analyses of clinical datasets in PD populations comparing different sensors, sensor placements, and algorithms have been conducted.

We here present clinical validation data of trunk‐ and wrist‐derived single sensor–based machine learning (ML) models from patients with PD collected in a standardized fashion.

This will provide information on the correlation of clinical and digital output data in this population, comparison of different sensory placements, and evaluation of clinical meaningfulness of such data. We used methodology according to and analogous to clinimetric validation methods for clinical rating scales to allow for an evaluation in the framework of assessment of clinical outcome measures [18]. It will also provide information for future studies and allow for more standardized reporting of such data. We hypothesized that there would be strong correlations between the trunk‐ and wrist‐worn device outputs and between both and corresponding clinical data. The trunk‐worn device data were expected to correlate most strongly with axial measures of PD and the wrist‐worn devices with assessments of limb movements. We also examined whether the outputs from the digital devices would correlate with functional and patient‐reported measures.

## 2. Materials and Methods

**FIGURE 1:**
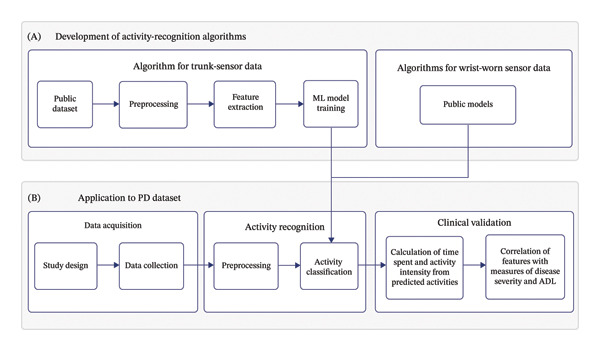
Overview of the study methodology.

Figure 1 provides a schematic overview of the study workflow. In brief, raw accelerometer data collected from trunk‐ and/or wrist‐worn devices were preprocessed and analyzed using one trunk‐based and two wrist‐based ML models to generate activity and movement‐related features. These sensor‐derived outputs were subsequently examined in relation to clinical and patient‐reported measures, including the MDS‐UPDRS and EQ‐5D‐5L, to assess their clinical validity in people with PD.

### 2.1. Study Design

Participants were recruited from two clinical trials with similar design: the PD‐Care Feasibility study, which tested the feasibility of recruitment and study methods ahead of a full randomized controlled trial of a facilitated self‐management intervention in PD [19], and the ADepT‐PD Feasibility study [20], which was a feasibility trial for an antidepressant trial for people with PD and depression. In both trials, participants were assessed by trained raters with clinical scales and questionnaires, including the MDS‐UPDRS and a health‐related quality of life measures, the EQ‐5D‐5L [21]. In both studies, participants were invited to participate in a substudy to assess the clinical usefulness of a wearable device to measure features of PD. The studies were approved by the London–Riverside Research Ethics Committee (REC number: 19/LO/0288) and the London–Queen Square Research Ethics Committee (REC reference: 18/LO/1470), respectively.

### 2.2. Data Collection

Participants who consented were sent Axivity AX3 wearable devices, 3‐axis accelerometers, with instructions on how to attach and wear the devices (including an instruction to wear them on the right wrist for the wrist‐worn devices) and how to return the devices. This device was chosen as it is a popular and low‐cost device providing raw data which has been used in several studies in people with PD [22]. The start and stop dates and the sampling rate (100 Hz) for recording were preprogrammed into the devices before being posted, and participants were instructed to begin and end wearing the devices at preprogrammed times. Data download was undertaken by the researchers upon the return of the devices. In the ADepT‐PD study, the sensor was worn on the trunk. In the PD‐Care Feasibility study, users were instructed to wear two devices simultaneously: an AX3 device both on the wrist and on the trunk. In both trials, the maximum duration of wear was 7 days. The devices were set to record at an (approximate) 100 Hz sampling rate.

### 2.3. Data Processing

The data from the Axivity AX3 was provided in .CWA format and was converted to .csv files using the open‐source software applicationOmGui before processing.

### 2.4. Development of the ML Models

For the trunk‐worn sensor data, a ML model was trained for activity recognition using two public datasets, HAR70 and HAR70+, which include trunk‐worn Axivity AX3 data in healthy controls containing 22 healthy adults, on average 38.6 ± 14 years old and 18 older adults aged 79.6 ± 7.6. The trunk acceleration data were preprocessed, segmenting them into 10 s periods. The 3‐axis data were resampled to a consistent 50 Hz for each signal to correct errors in the sensors’ clock, and the high‐frequency noise was filtered out using a 4^th^‐order Butterworth filter at 20 Hz. The Euclidean norm of the 3‐axis acceleration was computed to calculate the acceleration magnitude.

Activity labels were grouped into lying down, sitting/standing, walking (including walking upstairs and downstairs), and other activities (such as running or cycling). For classification, features including statistical measures of signal acceleration, body orientation, and continuous wavelet transform features were extracted. A random forest (RF) classifier was trained to classify activities in 10‐s time windows using 36 features selected from an initial set of 70 features. Activity levels for the trunk model were grouped in a similar way as for the grouping provided by the wrist‐based models, which correspond to widely available activity levels to provide interpretable and comparable information and allowed direct comparison of activity patterns derived from different sensors and models. Model evaluation was conducted using leave‐one‐participant‐out cross‐validation. The technical details of the ML model development are reported elsewhere (manuscript in preparation).

For the wrist‐worn sensor data, ML models that are publicly available (Biobank Accelerometer Analysis) could be directly applied. They allow prediction of activities from accelerometer data, generating time series and summary metrics of different activities [16, 17]. We here used two models: ModelWrist1, which predicts six predefined classes of behavior: bicycling, sit/stand, walking, vehicle, mixed activity, and sleep, and ModelWrist2, which predicts activity intensities, including for sleep, sedentary, light activity, and moderate‐to‐vigorous intensity activity. Categories were further grouped into sleep, sit/stand, walking, and “other activities.” This harmonized outputs across models with different classification schemes, enabling direct comparison between the self‐trained trunk model and publicly available wrist models.

### 2.5. Application of Models To the PD Dataset to Predict Activity Levels

**FIGURE 2:**
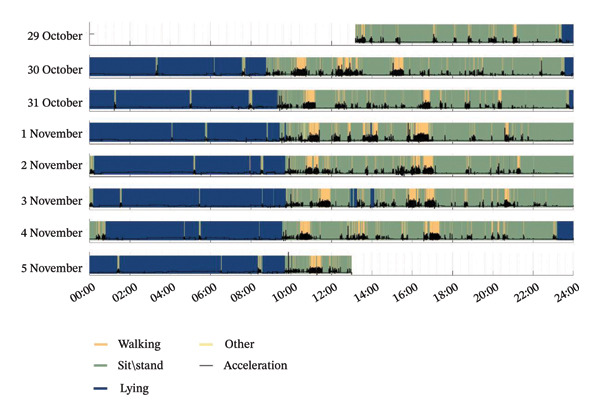
Predicted human activity patterns on Parkinson’s disease patients. Example of visualization plot for activities during a 7‐day period as predicted by the trunk sensor–worn model for one participant. Different colors indicate different types of activities.

The PD dataset was first preprocessed in the same way as the public dataset to ensure accurate activity predictions in the new data. The trained model for the trunk data and the public models for the wrist data were then applied to predict activities. An example of predicted activity patterns of a PD patient is shown in Figure 2. Activity labels and signals were segmented into 24‐h chunks from 12 p.m. to 12 p.m. for each participant. Chunks that did not reach 24 h were excluded from the analysis. Activity‐related features derived according to the labels predicted by ML models were generated for all individuals using wrist and trunk data for a 24‐h period. We calculated the percentage of time spent in each of the different activity levels, including lying time separately during the night (10 p.m. to 10 a.m.) and during the day, and statistical measures of acceleration intensity of the categories (mean, standard deviation, median, and variability). During walking periods, heel strikes and step durations were extracted. Heel strikes are defined as the largest peaks in acceleration magnitude, and step time is the interval between two peaks. Steps longer than 2.5 s were excluded as they may indicate rest periods, as were step counts of less than five between any two gaps, as they may not represent continuous walking, thus skewing variability and walking characteristics.

To avoid overestimating correlations due to multiple data segments from the same participant and varying recording lengths for different participants, data features from each participant were averaged across several 24‐h chunks.

### 2.6. Correlation of Activity and Activity Severity With Clinical Features and Function

Duration and acceleration intensity of extracted activity features were correlated with severity levels of clinical features hypothesized to measure corresponding clinical constructs. These included items on the MDS‐UPDRS Part II (motor aspects of experiences of daily living), Part III (motor examination), and Part IV (motor complications).

Several items from the MDS‐UPDRS Part III were excluded from the correlation analysis due to missing data owing to the remote nature of assessments (rigidity and postural stability; lower limb rest tremor, toe tapping, and leg agility [in PD‐Care only]; and gait and freezing of gait [in ADepT‐PD only]). Missing values of individual items were imputed by using the average score of the available MDS‐UPDRS Part III subscores for the participant. For all analyses involving the MDS‐UPDRS Part III total score, the “MDS‐UPDRS Part III” total score reported throughout this paper refers to a modified total score computed by summing scores for items available for all participants.

For the trunk‐worn sensor, the scores of items assessed separately on the left and right were averaged. For the wrist‐worn data, the scores from the right hand were used (as this corresponded with the wear instructions to participants). In addition, we correlated the sensor‐derived features with relevant total and functional scores, including the Hoehn and Yahr stage; MDS‐UPDRS Parts II, III, and IV domain scores; and the EQ‐5D‐5L index and visual analog scale scores.

### 2.7. Statistical Analysis

Correlations between the activity features of the models and clinical features were examined using Kendall’s Tau and Spearman’s rank correlations. As there was strong agreement between the results, we here report the Spearman correlation results. The strength of correlations was grouped into strong correlations (*r* ≥ 0.7), moderate correlations (*r* ≥ 0.4), and weak correlations (*r* ≥ 0.1).

## 3. Results

Ten people with PD agreed to participate, and the sensor data obtained are valid; six were recruited from the PD‐Care Feasibility study and four from the ADepT‐PD feasibility study. There were data for the trunk‐worn device from eight participants and wrist‐worn device data from six participants available for analysis. The clinical data of the participants are given in Table 1. Overall, 27 sets of 24‐h data were from trunk sensor data and 22 sets from wrist sensor data.

**TABLE 1:** Demographic data and clinical scores for participants.

Participants	F23	F27	F05	F16	F19	F18	A07	A09	A01	A06
Gender	Male	Male	Male	Female	Female	Male	Male	Male	Male	Male
Age	45	70	82	69	80	77	41	53	52	77
MDS‐UPDRS Part I	17	20	29	27	45	21	8	6	22	11
MDS‐UPDRS Part II	15	33	33	38	41	15	8	10	12	5
MDS‐UPDRS Part III	29	43	46	47	47	31	3	15	10	8
MDS‐UPDRS Part IV	10	13	17	19	17	6	0	7	11	2
EQ‐5D‐5L_mobility	1	2	1	3	2	1	2	1	3	1
EQ‐5D‐5L_self‐care	1	1	1	2	3	1	1	2	2	1
EQ‐5D‐5L_activities	1	2	3	3	4	1	2	2	2	1
EQ‐5D‐5L_pain	2	1	1	3	4	1	1	2	3	2
EQ‐5D‐5L_anxiety	2	1	1	2	3	2	3	1	2	2
EQ‐5D‐5L_health	84	30	50	50	28	69	65	80	60	80

*Note:* The MDS‐UPDRS Part III total scores excluded the items rigidity, postural stability, gait, freezing of gait, toe tapping, leg agility, and rest tremor amplitude for the lower extremities.

### 3.1. Average Daily Activity Levels

**FIGURE 3:**
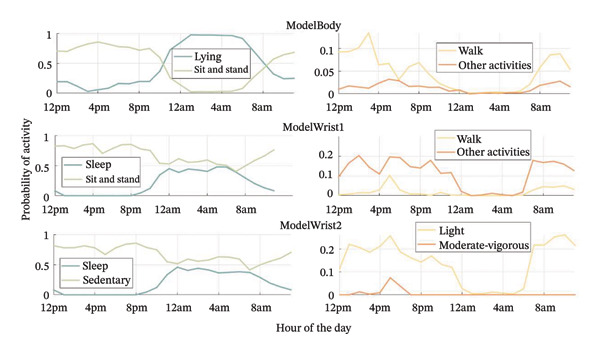
Averaged probabilities of different activities across participants during the day predicted by different models, trunk‐worn sensor‐derived (ModelBody) and wrist‐worn sensor‐derived (ModelWrist1 and ModelWrist2).

Activity level patterns over the 24‐h periods as identified in all three models were as expected for human activity, e. g., “Sleep/Lying” had a higher probability at night, and more activities (walking and other activities) were predicted during the daytime, and more activities were predicted in the morning and afternoon than in the evening. However, all three models suggest a relatively low level of activity of the participants during the daytime, i. e., mostly “Sitting/standing” rather than greater activity, likely reflecting reduced activity levels in PD patients. The probability of each activity level, aggregated from different 24‐h sets from all participants, is shown plotted against the time of day in Figure 3.

In those who used both trunk‐worn and wrist‐worn sensors, the percentage of time spent in different activities during the day, as an indicator of overall mobility and possibly on‐ and off‐time, was similar between trunk‐ and wrist‐worn devices. However, whilst the trunk‐worn sensor model predicted “Lying” during the night with nearly 100% probability, both wrist models predicted “Sleep” at around 50% during the night, with the other half predicted as “Sit‐stand/Sedentary” activity. Further analysis showed that two participants were classified as having a very low probability of “Sleep” at night with both wrist‐based models (on average, 2 h and 3 h per night). In contrast, the trunk model predicted one of these two participants to have a high probability of “Lying” from 11 p.m. until 10 a.m., and the other to have a longer duration of “Lying” during the night using the trunk‐based model than “Sleep” using the wrist‐based model. This indicates that they had additional movements in their limbs whilst lying down. Similarly, there was also a higher possibility of “Lying” predicted by the trunk model during the daytime, but almost no possibility of “Sleep” predicted by the wrist models. A detailed summary of these observations is provided in Table 2.

**TABLE 2:** Activity duration predictions (24‐h average) for different participants derived from trunk and wrist models.

	**Participant**	**Lying (night) (h)**	**Lying (daytime) (h)**	**Sit or stand (h)**	**Walking (h)**	**Activity (h)**

Trunk model	F23	8.9 ± 0.1	1.7 ± 0.1	4.3 ± 1.2	2.6 ± 1.4	4.6 ± 1.4
	F27	11.9 ± 0.7	2.2 ± 0.9	5.6 ± 0.9	0.3 ± 0.1	0.4 ± 0.1
	F05	11.8 ± 0.9	1.1 ± 0.8	6.2 ± 0.9	0.4 ± 0.4	1.0 ± 0.6
	F16	7.9 ± 0.6	2.8 ± 0.2	4.9 ± 0.0	0.4 ± 0.1	0.6 ± 0.4
	A07	8.9 ± 0.3	0.5 ± 0.2	6.2 ± 0.4	1.3 ± 0.2	1.6 ± 0.3
	A09	8.2 ± 1.2	0.5 ± 1.0	6.6 ± 1.9	1.7 ± 1.6	1.9 ± 1.9
	A01	6.2 ± 1.1	0.5 ± 0.6	5.9 ± 0.5	1.6 ± 0.6	3.2 ± 0.9
	A06	9.4 ± 0.4	0.0 ± 0.1	6.9 ± 0.4	2.2 ± 0.3	2.2 ± 0.3

	**Participant**	**Sleep (night) (h)**		**Sedentary (h)**	**Walking (h)**	**Activity (h)**

Wrist model	F23	8.0 ± 0.0		5.2 ± 0.4	2.1 ± 1.2	2.6 ± 0.5
	F27	8.6 ± 1.5		6.8 ± 2.5	0	0.1 ± 0.1
	F05	3.9 ± 1.8		5.6 ± 2.5	0.2 ± 0.2	0.9 ± 1.1
	F16	3.0 ± 0.8		7.2 ± 0.9	0	0.2 ± 0.1
	F19	1.9 ± 1.7		4.9 ± 1.2	0.1 ± 0.1	3.1 ± 1.2
	F18	7.6 ± 0.2		4.6 ± 3.7	0.6 ± 0.2	0.7 ± 0.3

### 3.2. Correlation of the Sensor‐Derived Model Data With Clinical Data

#### 3.2.1. Trunk Sensor Data

##### 3.2.1.1. Correlations With Clinical Features of PD

There were strong correlations of various activity features extracted from the trunk sensor, particularly time spent lying down during the day, step duration variability, and walking and other activity acceleration with axial MDS‐UPDRS Part III items, such as those measuring impairment getting up from a chair, walking, posture, facial expression and body bradykinesia (Figure 4B,C). There were also strong correlations with overall disease severity scores, including Hoehn and Yahr scores, reflecting the fact that Hoehn and Yahr staging is a motor staging system concentrating on axial features, and the MDS‐UPDRS Part III total score (Figure 4A). Similarly, there were strong correlations with the MDS‐UPDRS Part IV items, time in off‐state and complexity of motor fluctuations (Figure 4D).

FIGURE 4Correlations of activity features from the trunk‐worn sensor‐derived model with (A) measures of overall function, (B) MDS‐UPDRS Part II items, (C) MDS‐UPDRS Part III items, (D) MDS‐UPDRS Part IV items, and (E) MDS‐UPDRS Part III lower limb items in a subset. ^∗^Statistical significance: *p* value < 0.05; ^∗∗^ *p* value < 0.01.

**Figure:**
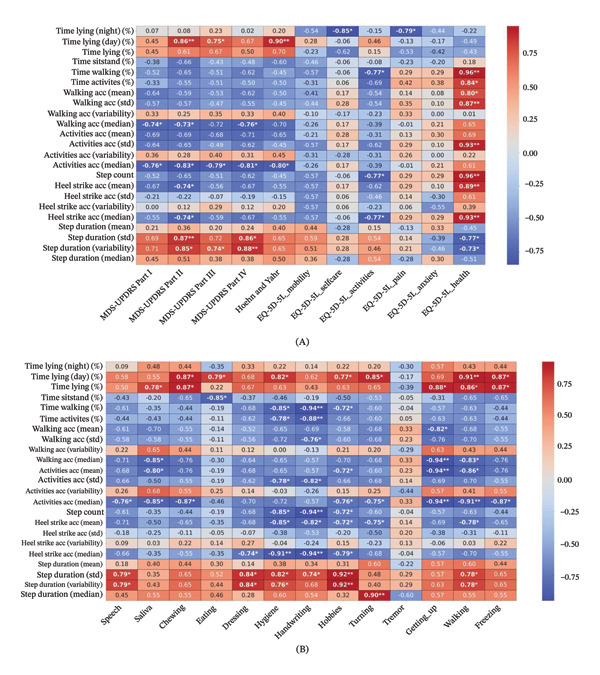

**Figure:**
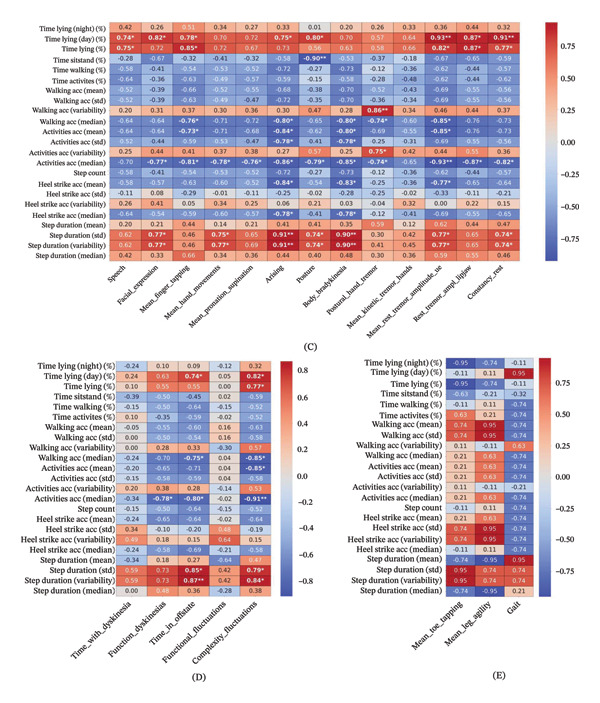

##### 3.2.1.2. Correlation of Wearable Sensor Data With Daily Functioning

The trunk sensor‐derived output features also correlated strongly with functional scores, and individual items on the MDS‐UPDRS Part II except tremor, and with the patients’ assessment of general health on the visual analog scale of the EQ‐5D‐5L (Figures 4A,B). Similar to the correlations with individual MDS‐UPDRS Part III features, the model’s output features of median acceleration of walking and of other activities, of time spent lying down during the day, and of variability of step duration were most frequently and strongy correlated with these scores.

#### 3.2.2. Wrist Sensor Data

##### 3.2.2.1. Correlations With Clinical Features of PD

The correlations of the wrist‐worn sensor data outputs were similar to those of the trunk‐worn but weaker, except for correlations with postural tremor and rest tremor constancy (Figures 5C,D). Correlations with MDS‐UPDRS total scores and Hoehn and Yahr scores were also similar to the trunk‐worn sensor output data but generally weaker.

FIGURE 5Correlations of activity features from the wrist‐worn sensor‐derived model with (A) measures of overall function, (B) MDS‐UPDRS Part II items, (C) MDS‐UPDRS Part III items, and (D) MDS‐UPDRS Part IV items. ^∗^ *p* value < 0.05; ^∗∗^ *p* value < 0.01. Activity features highlighted in purple are derived from ModelWrist2, features highlighted in green are derived from a combination of ModelWrist2 and ModelWrist1, while all other features correspond to ModelWrist1.

**Figure:**
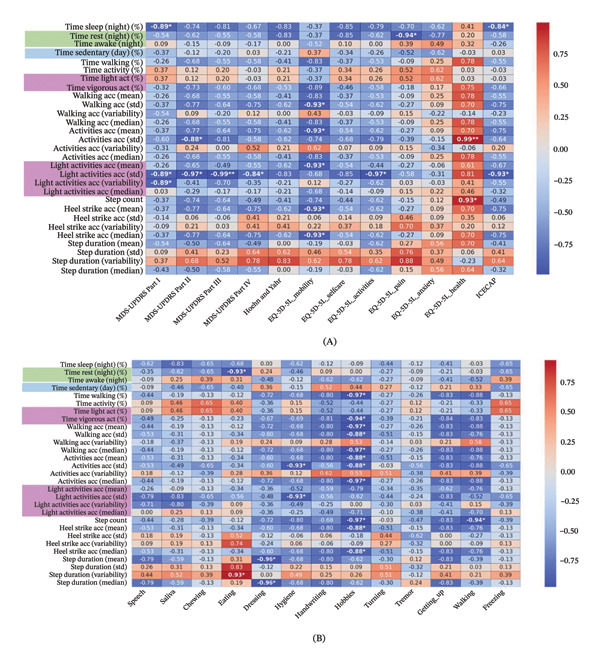

**Figure:**
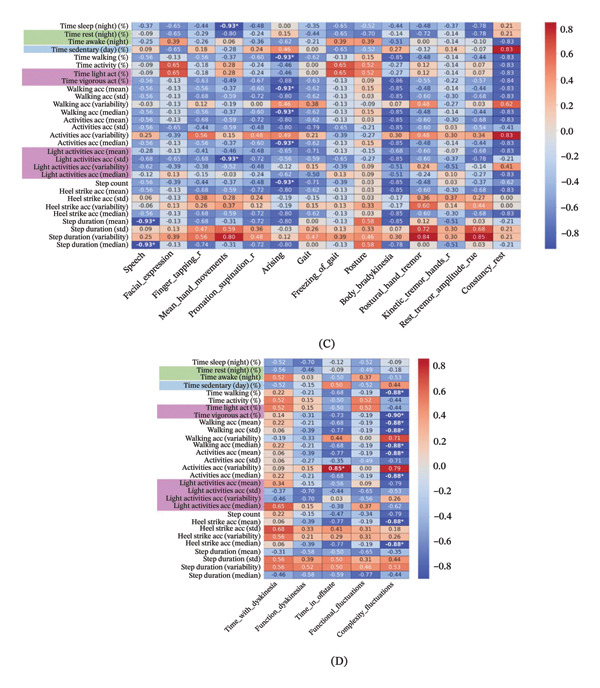

##### 3.2.2.2. Correlation of Wearable Sensor Data With Daily Functioning

For the MDS‐UPDRS Part II items, the correlations of wrist‐worn output data were generally weaker, especially with axial features such as freezing and turning, but slightly stronger with others such as eating and undertaking hobbies. Correlations were stronger for the wrist‐worn sensor data than the trunk‐worn device withmost of the EQ‐5D‐5L domains, particularly with acceleration of heel strike, walking, and other activities, and for the EQ‐5D‐5L pain domain with nighttime rest, and there were similar correlations to the trunk‐worn sensor data with patients’ assessment of their general health state (Figure 5 for Model 2).

### 3.3. Sensitivity Analysis on Sleep‐Related Features

In order to investigate the discrepancy between “Sleep” and “Lying down” time between the wrist and trunk models, we undertook a post hoc sensitivity analysis of the correlation between sensor output data and the MDS‐nonmotor symptom scale’s sleep item scores, which were available only in the PD‐Care study. Time lying from the trunk‐worn sensor data correlated moderately with insomnia and dozing during the day but not RBD, RLS, PLM, or breathing difficulties, whereas sleep time during night using the wrist data also strongly correlated with RBD and PLM scores (data not shown).

## 4. Discussion

This is one of the first studies exploring the relationship between clinical features of PD as assessed on the gold‐standard rating scale MDS‐UPDRS and single sensor–derived output data in patients with PD in a real‐world setting. We found that both trunk‐worn as well as wrist‐worn sensor data have strong and clinically meaningful correlations with clinical data measuring the severity of features of PD. Our results are comparable with other studies in the literature on predicting individual MDS‐UPDRS motor scores using single or different multisensor combinations, and expand these to include correlations of MDS‐UPDRS items with activity levels as well as correlations of gait characteristics and to include correlations of these along with the overall function [7, 23, 24].

Models based on both the trunk‐ and wrist‐worn data identified the main activities, e. g., time lying down, walking, and other activities, and the results were similar using both body locations and different ML models. However, there was a discrepancy in the classification of time spent in sleep in the wrist sensor model and lying down in the trunk‐worn model. This may be due to the slightly different definitions of the labels “Lying” and “Sleep” in the models, i. e., “Lying” indicates a state of lying down without necessarily sleeping. However, it is also possible that periods with PD‐related limb movements, e. g., due to periodic limb movements or REM‐sleep behavior disorder, were incorrectly not classified as “Sleep” in the models derived from wrist‐worn sensor data. This is supported by the finding that the overall sleeping time for some participants was unrealistically low in the classification by the wrist‐based models (less than 5 hours across participants; see Table 2). Furthermore, a post hoc sensitivity analysis suggested that whilst time lying down using the trunk sensor data was correlated with insomnia and dozing during the day, sleep time during the night was associated primarily with RBD and PLM movements, which were detected using a wrist‐worn model.

There is considerable variability in the reporting and methodology in this field, but our study’s methodology aligns with clinical validation studies of other measurement methods such as clinical scales and patient‐reported outcome measures [18, 25]. Such an approach can help standardize and allow for comparisons between different approaches and studies.

The clinical correlations of the sensor‐derived measures suggest that single wearable sensors do measure constructs important in the assessment of severity of PD according to the current gold standard, providing evidence for the validity of such assessments as tools to assess severity of PD in patients with PD. However, there are also clear differences between such sensor‐derived measures and clinical measurements. Sensor‐derived measures provide a more continuous recording over several days, and single sensor measures reflect movement of one body part, reflected in the difference between trunk‐ and wrist‐worn sensor clinical correlations. The majority of the findings are in keeping with a priori assumptions, i. e., trunk‐worn sensors provide strong correlations particularly with axial features such as posture, walking, and body bradykinesia, but also with limb bradykinesia and motor fluctuations, and wrist‐worn sensors provide stronger correlations with postural tremor and constancy of rest tremor, together with the ability to perform hobbies and eat. These differences between results from trunk‐ and wrist‐worn sensors should be taken into account when comparing results from sensor‐derived measures both in clinical practice and trials. Whilst sensors on both body locations can capture functionally relevant disease features, future trials should consider using a trunk‐worn sensor, if practically feasible, in studies where axial features are most relevant, and wrist‐worn sensors in studies where tremor and activities requiring hand function are most relevant.

It was slightly surprising that limb bradykinesia features did not correlate more strongly with the wrist‐worn sensor data, particularly in the upper limbs. This suggests that limb bradykinesia, which is defined by slowness, fatiguing, and interruptions in distal limb movement, is not well captured by the sensors, whereas the greater movements of complex dyskinesias, which are typically more proximal, are better captured. Thus, distal movements may be better captured using active or passive measurements of finger dexterity such as tapping tasks or instrumental gloves.

Of note, for both body locations, there were strong correlations with measures of activities of daily living, as indicated on the MDS‐UPDRS Part II and overall health on the visual analog scale, indicating the functional relevance of these sensor‐derived measures. This was also reflected in correlations with EQ‐5D‐5L domain scores, and these correlations appeared stronger for the wrist‐worn sensors. This suggests that the single sensor assessments are valid for the assessment of severity and functional impact of PD features and may be useful for supporting assessments in clinical trials and clinical practice.

The study has limitations, in particular the small size, imputation of missing clinical data for some items due to the remote assessments necessitated by the COVID pandemic, and the reliance on publicly available algorithms for the wrist‐worn measures. Although Spearman’s rank correlation is relatively robust in small samples, these findings need cautious interpretation. Further validation in larger cohorts spanning the full spectrum of disease severity is therefore recommended. In addition, future studies may further improve the information from sensor‐derived measures by providing more comprehensive algorithms or additional sensor placements, including other active or passive movement measurements.

## 5. Conclusion

This study provides novel validation data based on the correlation of output data from a single wearable device and in‐depth clinical assessments in patients with PD, with some comparative data between wear in different body parts, which suggest that single sensor‐derived measurements using both trunk‐ and wrist‐worn single sensors are valid assessments in people with PD. The strong correlation of these outputs not only with relevant clinical measurements but also with function and health status supports the validity of the digital output measures from a widely available device analyzed using publicly available algorithms. The integration of continuous sensor‐derived data with other clinical assessments or biomarkers has the potential to provide a more holistic view of PD severity and provide sensitive, clinically relevant and continuous information on severity of PD symptom severity in clinical practice and trials. However, prospective, larger and more inclusive studies are required to confirm these findings. Furthermore, methodologies and reporting in this field should be standardized to facilitate comparisons between studies.

## Author Contributions

Yanke Sun: methods design related to engineering aspects, data analysis, generation of figures and tables, drafting of part of the Methods and Results, critical revision, and editing of all manuscript versions. Sally Day: providing underpinning ideas and method design related to engineering aspects. Kate Walters: participant recruitment, data collection, and editing of the final manuscript.

Anette Schrag: co‐conceptualization and study design, oversight of data collection, writing of the paper, and critical revision and editing of all manuscript versions.

## Funding

This work was supported by the National Institute for Health and Care Research program grant RP‐PG‐1016‐20001.

## Disclosure

Financial Disclosures for the previous 12 months:

Yanke Sun: The author has no financial interests or relationships to declare in any of the following categories for the preceding 12 months.

Anette Schrag received research funding or support from University College London, National Institute of Health (NIHR), National Institute for Health Research ULCH Biomedical Research Center, the International Parkinson and Movement Disorder Society (IPMDS), the European Commission, and Parkinson’s UK. Anette Schrag also received honoraria for consultancy from Biogen, AbbVie, BIAL, and Otsuka and royalties from Oxford University Press.

## Ethics Statement

The studies were approved by the London–Riverside Research Ethics Committee (REC number 19/LO/0288) and the London–Queen Square Research Ethics Committee (REC reference: 18/LO/1470), respectively.

Written informed consent was obtained from all participants involved in this study.

We confirm that we have read the Journal’s position on issues involved in ethical publication and affirm that this work is consistent with those guidelines.

## Consent

Please see the Ethics Statement.

## Conflicts of Interest

The authors declare no conflicts of interest.

## Data Availability

The raw sensor datasets used in this study are available from the corresponding author upon reasonable request. All analysis code and scripts are openly accessible on GitHub: https://github.com/khorinaj/PDCare-ParkinsonsDisease-AccelerationAnalysis/tree/main.
